# In Design of an Ocean Bottom Seismometer Sensor: Minimize Vibration Experienced by Underwater Low-Frequency Noise

**DOI:** 10.3390/s18103446

**Published:** 2018-10-13

**Authors:** Xiaohan Wang, Shengchun Piao, Yahui Lei, Nansong Li

**Affiliations:** 1 Acoustic Science and Technology Laboratory, Harbin Engineering University, Harbin 150001, China; wangxiaohan@hrbeu.edu.cn (X.W.); leiyahui@hrbeu.edu.cn (Y.L.); linansong@hrbeu.edu.cn (N.L.); 2 Key Laboratory of Marine Information Acquisition and Security, Harbin Engineering University, Ministry of Industry and Information Technology, Harbin 150001, China; 3 College of Underwater Acoustic Engineering, Harbin Engineering University, Harbin 150001, China

**Keywords:** OBS (Ocean Bottom Seismometer), COMSOL, vibrational response, acoustic waves

## Abstract

Ocean Bottom Seismometers (OBS) placed on the seafloor surface are utilized for measuring the ocean bottom seismic waves. The vibration of OBS excited by underwater noise on its surface may interfere with its measured results of seismic waves. In this particular study, an OBS was placed on the seabed, while ray acoustic theory was used to deduce the sound field distribution around the OBS. Then using this information, the analytical expression for the OBS vibration velocity was obtained in order to find various factors affecting its amplitude. The finite element computing software COMSOL Multiphysics^®^ (COMSOL) was used to obtain the vibration response model of the OBS which was exposed to underwater noise. The vibration velocity for the OBS calculated by COMSOL agreed with the theoretical result. Moreover, the vibration velocity of OBS with different densities, shapes, and characters were investigated as well. An OBS with hemispherical shape, consistent average density as that of the seafloor, and a physical structure of double tank has displayed minimum amplitude of vibration velocity. The proposed COMSOL model predicted the impact of underwater noise while detecting the ocean bottom seismic waves with the OBS. In addition, it provides significant help for the design and optimization of an appropriate OBS.

## 1. Introduction

An Ocean Bottom Seismometer (OBS) is a kind of vibration observation instrument working on the seafloor and can be applied for observing natural earthquakes, as well as detecting artificial marine seismic waves [1]. The OBSs are smart and self-processing device which has replaced larger arrays and mooring ropes and hence has made regular marine seismic reflection exploration relatively easier. Similarly, they have a unique feature of lying on seafloor due to their weight. By lying stationary on the seafloor, the OBSs measure not only the underwater acoustic waves but seismic waves as well. These seismic waves may comprise of abundant longitudinal waves, converted shear waves, and multiple varieties of effective waves. Therefore, the OBSs can process the inversion method for exploration of seafloor internal structures and stratum resolution enhancements. For this particular reason, they have been extensively employed in geophysical investigations, detection of natural earthquakes, and offshore oil and gas explorations [2,3,4,5].

During the 1930s, the OBSs found their initial applications for the study of geological structures and deep-water explorations. Currently, a rudimentary node is still present at the SEG museum in Norman Oklahoman [6]. In 1966, Texas Instruments (US) utilized their self-made OBSs for long-time marine observations in offshore waters from Kuril Islands to Kamchatka. During this time, Scripps Institution of Oceanography also succeeded in developing their OBSs and applied them to test the seafloor pulses. Similarly, Moscow State University used their developed OBS to observe earthquakes in the Indian Ocean. Although the OBS technology was capable of playing an important role in exploiting oceanic deep structures and oil gas explorations, which was developed only in the technologically advanced countries. Before 2003, China had completed many times of submarine earthquake detections around the north continental margin of the South China Sea. All of these efforts were accomplished by international and regional cooperation, and none of the employed OBS was developed indigenously by China. China started the development of the OBSs relatively late, however, under the strong support of National 863 Program and specialized instruments development, multiple scientific institutions have developed technologically advanced OBSs. Thus, China has established itself to develop and manufacture its own reliable OBSs recently and has been involved in using OBS technology extensively for seafloor structure observations in the Bohai Sea, the South China Sea, and Southwest Indian Ocean. These successes have demonstrated that China has indigenously developed advanced OBS from principle prototypes [7,8].

**Figure 1:**
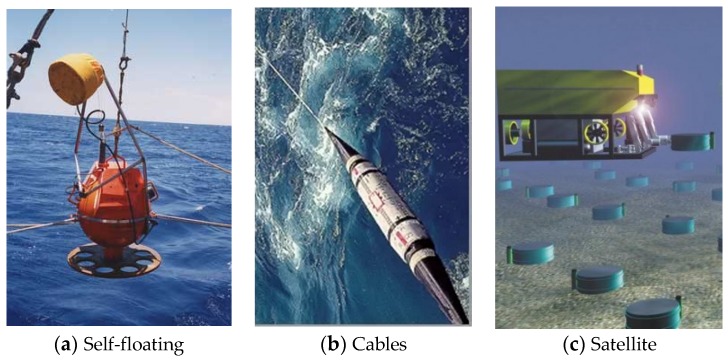
Various types of Ocean Bottom Seismometers (OBSs) placed on the seafloor.

According to application and apparatus variety, the OBSs can be classified into moored, self-floating (Figure 1a), cable (Figure 1b), and artificial satellite (Figure 1c) types. The moored OBSs are usually deployed in shallow water for convenient operations and recovery. The self-floating OBSs are applied in deep sea. They appear on surface for recovery due to positive buoyancy, which is obtained by releasing the weights after its work. The cable and artificial satellite types are usually applied in large numbers for seafloor geological explorations. In addition, large ships are required to deploy these OBSs along with their cables and underwater Remotely Operated Vehicles (ROV) for accurate placements [9,10,11]. The Table 1 below shows the comparative of present OBSs around the world.

The OBSs can be classified into short-period and long-period, depending on the frequency of received signal. The working frequency of long-period OBSs ranges from 0.01 to 10 Hz with a lifetime of few months to a couple of years. Analysis of this low frequency seismic data helps to measure submarine earthquakes as well as the movement of both crust and continental plates. The working frequency for short-period OBSs is 10 Hz~1 kHz with a lifetime of few days to a couple of weeks. These OBSs use the submarine slight shock excited either by air gun or some other artificial sound sources to achieve accurate seafloor geological explorations. In addition, they are used to detect marine resource such as oil, manganese nodule, and other mines [12,13].

In shallow water, the OBSs data is contaminated by various amounts of noises. The vertical vibrational velocity noise (later will be called ‘Vz noise’) depends strongly on the condition of the ocean bottom, with muddy bottoms generating strong noise and sandy bottoms giving almost noise free data. After different OBS configurations have been tested, we conclude that the ‘Vz noise’ is not dependent of the recording station configuration and instead is a true measurement of the vertical movement of the ocean bottom [14].

**Figure 2:**
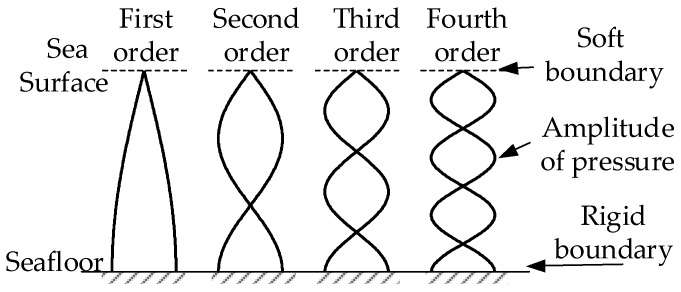
Amplitude of the pressure of normal mode wave of plane wave guide in shallow water on the wave guide cross section.

In addition to ‘Vz noise’, the OBSs recoded data is perturbed by the ocean water noise. For plane acoustic waves propagating in shallow water, the sea surface is approximately the soft boundary where the sound pressure is 0 and the reflection coefficient is −1. The seafloor approaches the rigid boundary where the normal (vertical) vibrational velocity is 0 and the reflection coefficient is 1. According to the normal mode theory, the amplitude of the pressure in each-order normal mode wave reached its maximum near the seafloor and reached its minimum near the surface, as is shown in Figure 2 [15,16,17]. 

**Figure 3:**
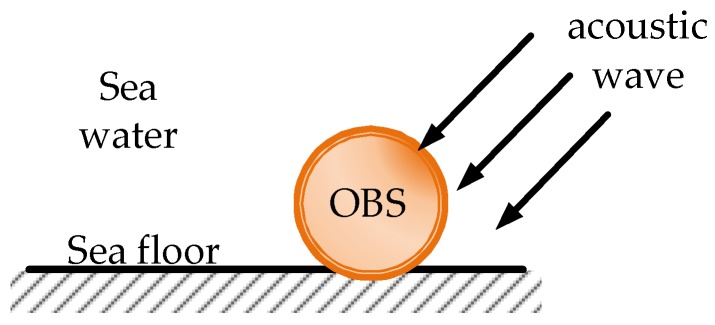
The acoustic wave around the OBS placed on the seafloor.

When the OBS is planted on the seafloor for acquiring seismic waves from natural or artificial sources (as is shown in Figure 3), it experienced the micro vibration by the acoustic waves on its surface. Thus, the vibration signal collected by the OBS not only includes the vibration of seafloor, but also involves the interfering signal of underwater noise. Based on the above findings, we need to develop a highly sensitive OBS which is capable of suppressing interfering signal from underwater noises, especially for shallow water applications.

The finite element method (FEM) is a numerical method for solving problems of engineering and mathematical physics. Typical problem areas of interest include structural analysis, heat transfer, fluid flow, mass transport, and electromagnetic potential, especially for multi-physics coupling. FEM subdivides those large problems into smaller, simpler parts that are called finite elements. The solution of these finite elements are assembled into a solution of the model. Recently, FEM is finding wide applications in both numerical and engineering calculations [18]. 

Various finite-element software has been extensively used for the calculations of sound fields and structural vibrations. In 1940s, finite element originated in applications for aeronautical and civil engineering in the field of structural analysis. It rapidly expanded to various engineering fields such as mechanics, physics, and mathematics, especially after the boom in the development of computer technology. It exhibits significant advantages such as high efficiency and accuracy while dealing with the multi-physics coupling. Scientific institutes have been using commercial finite element software to calculate the sound fields among various interfaces. Their results from simulation and experimental measurements suggested that FEM can calculate the sound field distributions in the presence of an interface both efficiently and accurately.

In this paper, the analytical expressions for both incident and scattering acoustic waves on the surface of OBS are deduced (Equations (12) and (13)) in the presence of an interface. Then, the equation for vibration velocity of OBS is derived (Equation (15)) from the sound pressure on its surface. COMSOL Multiphysics^®^ (COMSOL) is used for developing the vibration response model. This model calculates the vibration responses of the OBS experienced the low frequency (1 Hz~1 kHz) noise in water. Accuracy of this model is validated by comparing the COMSOL calculation with the theoretical calculation. Then, this particular model is utilized for measuring the vibration velocities of the OBSs for various material densities, shapes, physical structures, density gradients, and multi-variety casings. The OBS with hemispherical shape, consistent average density as that of the seafloor, and a physical structure of double tank has displayed minimum amplitude of vibration velocity. Our self-built COMSOL model reduces the interference of underwater noise on OBS signal. Finally, the experiment is carried out with our double tank OBS made up of cylindrical titanium alloy.

## 2. Vibration Velocities of the OBSs Placed on the Seafloor

**Figure 4:**
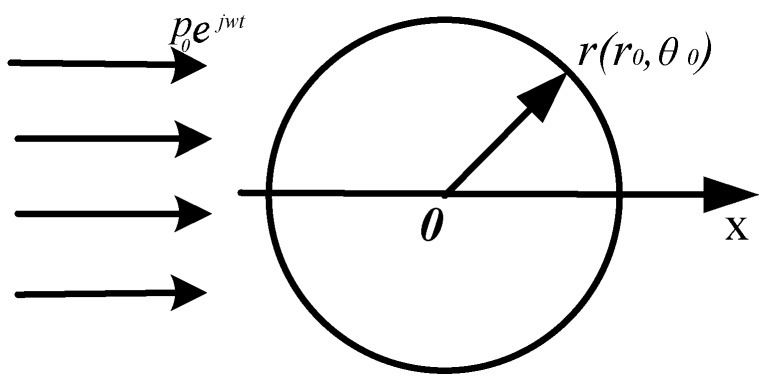
2-D axial symmetry schematic diagram of the spherical OBS in free field.

The vibration velocity response of a spherically solid iron OBS with diameter of *r*_0_ = *a* = 0.1 m in free field is analyzed initially. Figure 4 represents a simplified model where the incident wave is a plane wave *p*_0_*e^jwt^*, and the sound pressure at any point on the surface *S*_0_ of the OBS is [19].

(1) $$\;{p\;=\;p}_{i}{\;+\;p}_{s}\;$$

At any point in the spherical coordinate system, *p* is the total sound pressure, *p_i_* and *p_s_* are the sound pressures of incident and scattering waves. If the time factor *e^jwt^* is ignored, the expression of *p_i_* and *p_s_* at any point in free field can be written as follows as mentioned in reference [19]:

(2) $$\;p_{i}{(r,t,\theta \;)\;=\;p}_{0}e^{j\omega t}\overset{\infty }{\underset{m\;=\;0}{\sum }}{\left(-j\right)}^{m}\left(2m\;+\;1\right)P_{m}{(\cos\theta )j}_{m}\;\left(kr\right)\;$$

(3) $$\;p_{s}{(r,t,\theta )\;=\;p}_{0}e^{j\omega t}\overset{\infty }{\underset{m=0}{\sum }}c_{n}P_{m}{(\cos\theta )h}_{m}^{1}\;\left(kr\right)\;$$

$$\;k\;=\;\frac{\omega }{c}\;$$

where, *p*_0_ is the amplitude of the incident sound wave, *w* is the angular frequency, *c* is the underwater sound speed, *t* is the time factor, *j* is an imaginary unit, *k* is the wave number, *j_m_* is an *m*-order Bessel Function, *h*^1^*_m_* is an *m*-order Hakel Function of the first kind, *P_m_* is an *m*-order Legendre function of the first kind*,* and *c_n_* is the coefficient determined by the boundary between the surface of OBS and the water [19].

For both adjacent inner and outer points of the surface of sphere, the boundary condition for elastic sphere is that:

(1) If normal stress is continuous, the sound pressure of outer point is equal to the normal stress of inner point but with opposite direction;
(2) If normal vibration velocity is continuous, the vibration velocity of outer point is equal to the vibration velocity of inner point in normal direction but with opposite direction;
(3) If tangential stress at both adjacent inner and outer points of the surface of sphere is zero, then the formula is expressed as [20]:

(4) $$\;\{\begin{array}{c} {T_{rr}|}_{r\;=\;a\;}=\;{{-(p}_{i}{+\;p}_{s})|}_{\;r\;=\;a\;}\\ {v_{1r}|}_{r\;=\;a\;}=\;-{v_{2r}|}_{\;r\;=\;a\;}{=\;v}_{x}\cos\theta \\ {T_{r\theta }|}_{r\;=\;a}\;={{\;T}_{r\varphi }|}_{r\;=\;a}\;=\;0 \end{array}\;$$

In Equation (4), the *T_rr_* and *T_rϑ_* are the normal and tangential stresses in radial directions, *v*_1*r*_, *v*_2*r*_ are the velocities in radial directions. Inserting the Equation (2) and (3) into Equation (4), the coefficient *c_n_* is:

(5) $$\;c_{n}=k{\left(-1\right)}^{n}\left(2n+1\right)h_{n}^{1}\left(kr_{0}\right)\sin\eta_{n}e^{-j\eta_{n}})\;$$

(6) $$\;\tan\eta_{n}\left(x\right)=\frac{-\left[j_{n}\left(x\right)F_{n}-krj\prime_{n}\left(x\right)\right]}{\left[u_{n}\left(x\right)F_{n}-kru_{n}\prime \left(x\right)\right]}\;$$

(7) $$\;F_{n}=\frac{\rho }{\rho_{1}}\frac{k_{2}^{2}a}{2}\frac{A_{n}{-B}_{n}}{D_{n}{-E}_{n}}\;$$

$$\;A_{n}=\frac{x_{1}{j^{\prime }}_{n}\left(x_{1}\right)}{k_{1}{j^{\prime }}_{n}\left(x_{1}\right)-j_{n}\left(x_{1}\right)};\;B_{n}=\frac{2\left(n^{2}+n\right)j_{n}\left(x^{2}\right)}{\left(n^{2}+n-2\right)j_{n}\left(x_{2}\right)+{\left(x_{2}\right)}^{2}j_{n}^{\prime \prime }\left(x_{2}\right)};\;$$

$$D_{n}=\frac{\left(x_{1}\right)\left\{\left[\frac{\sigma }{1-\sigma }\right]j_{n}\left(x_{1}\right)-j_{n}^{\prime \prime }\left(x_{1}\right)\right\}}{x_{1}{j^{\prime }}_{n}\left(x_{1}\right)-j_{n}\left(x_{1}\right)};\;E_{n}=\frac{2\left(n^{2}+n\right)j_{n}\left(x_{2}\right)x_{2}j_{n}^{\prime }\left(x_{2}\right)}{\left(n^{2}+n-2\right)j_{n}\left(x_{2}\right)+{\left(x_{2}\right)}^{2}j_{n}^{\prime \prime }\left(x_{2}\right)}\;$$

$$\;j_{n}^{\prime }\left(x\right)=\frac{d}{dx}j_{n}\left(x\right);j_{n}^{\prime \prime }\left(x\right)=\frac{d^{2}}{dx^{2}}j_{n}\left(x\right);\;$$

$$\;u_{n}^{\prime }\left(x\right)=\frac{d}{dx}u_{n}\left(x\right)\;\;x=ka;x_{1}=k_{1}a;\;x_{2}=k_{2}a;\;k=\frac{\omega }{c};\;k_{1}=\frac{\omega }{c_{1}};k_{2}=\frac{\omega }{c_{2}};$$

where, *c*_1_ and *c*_2_ are the longitudinal and transverse wave velocities of the iron ball, *ρ* is the water density, and *ρ*_1_ is the density of the OBS made up of iron. According to the Newton’s second law, the vibration velocity v of the OBS in free field is:

(8) $$\;v\;=\int \frac{\iint_{S_{0}}^{}(p)dS}{\frac{4}{3}{\pi r}^{3}\rho_{1}}\;$$

**Figure 5:**
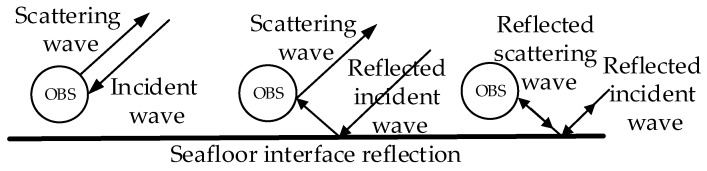
Incident and scattering acoustic waves of OBS.

The incident acoustic waves on the OBS can be divided into two parts as shown in Figure 5, namely the direct wave and the reflected incident wave through seafloor. The scattering waves can be divided into three parts [21,22,23]. The first scattering wave comes from the OBS in the free field. The second scattering wave is generated by the reflected incident wave from the seafloor and vice versa. The third scattering wave is generated from the reflected incident wave and then reflected by seafloor again.

**Figure 6:**
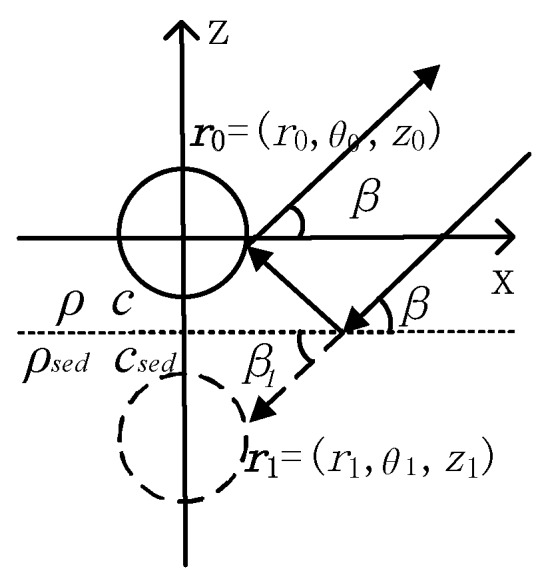
The acoustic wave around the spherical OBS placed on seafloor.

The OBS placed on the seafloor is shown in Figure 6. The *β* angle with seafloor is formed by the incident angle of acoustic wave. Here, the incident wave with interface is clearly different from Equation (2). According to reference [15]. The incident wave can be decomposed into:

(9) $$\;p_{\mathrm{inew}}{\;=\;p}_{i\beta \;}{+\;R(\beta )\;p}_{i(-\beta )}\;$$

where, *R* is the reflection coefficient as given in Reference [15]:

(10) $$\;R\left(\beta \right)\;=\;\frac{\rho_{1}c_{1}{\sin\beta -\rho \mathrm{csin}\beta }_{1}}{\rho_{1}c_{1}{\sin\beta +\rho \mathrm{csin}\beta }_{1}}\;$$

According to the Snell’s law:

(11) $$\;\frac{c}{\cos\beta }\;=\;\frac{c_{1}}{{\cos\beta }_{1}}\;$$

(12) $$p_{i\beta }=p_{0}\sum_{n=0}^{\infty }\sum_{m=0}^{n}q_{nm}P_{n}^{m}\cos(\varphi )j_{n}(ka)\cos(m\beta )\;$$

where, *q_mn_* is the parameter of the incident wave whose expression is suggested in Reference [21,22,23]. Then, the scattering wave formed by incident wave on OBS surface is:

(13) $$p_{s\beta }=p_{0}\sum_{n=0}^{\infty }\sum_{m=0}^{n}t_{n}h_{n}(ka)q_{nm}P_{n}^{m}\cos(\varphi )\cos(m\beta )\;$$

Though the scattering coefficient *t_n_* can be derived by boundary condition equation i.e., Equation (4), but for the sake of simplicity, the expression for scattering coefficient *t_n_* is obtained from Reference [21,22,23].

**Figure 7:**
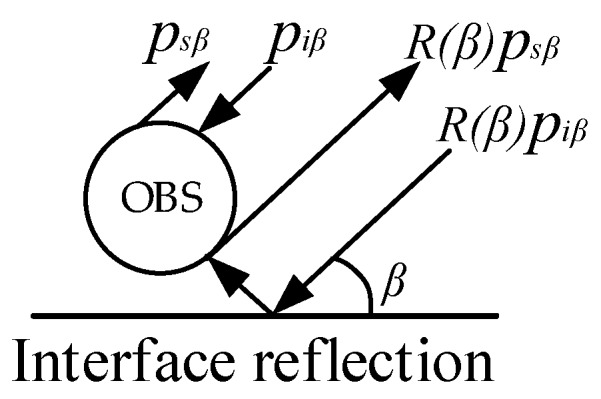
Acoustic wave on the surface of the OBS.

Four kind of acoustic waves on the surface of the OBS may be observed in Figure 7, which are: (1) The direct wave *p_iβ_*; (2) the reflected scattering wave *p_sβ_* formed by the direct wave; (3) the incident wave *R(β)p_iβ_* through the seafloor; and (4) the scattering wave *R(β)p_sβ_* formed by the reflected incident wave [21,22,23].

Then, the sound pressure *P_new_* and vibration velocity *v_new_* on OBS placed on the seafloor are:

(14) $$\;p_{\mathrm{new}}{\;=\;p}_{i\beta \;}{+\;p}_{s\beta \;}{+\;R(\beta )\;(p}_{i(-\beta )}+p_{s(-\beta )})\;$$

(15) $$\;v_{\mathrm{new}}=\int \frac{\iint_{S_{0}}^{}{(p}_{\mathrm{new}})\mathrm{dS}}{\frac{4}{3}{\pi r}^{3}\rho_{1}}\mathrm{dt}\;$$

The coefficient *t_n_*, *q_mn_* [21,22,23] in Equations (12) and (13) are determined by the physical parameters (especially for the material density), shapes, and characters of the OBS. Usually, the OBS shapes are of irregular geometries which makes the solution of analytical expression for its vibration velocity a bit difficult. For that reason, it needs to be accomplished through other calculation methods as discussed in the Section 3.

## 3. COMSOL Model and Its Calculations

COMSOL Multiphysics^®^ is the general-purpose platform software for modeling engineering applications. We can use the core package on its own or expand its functionality with any combination of add-on modules for simulating designs and processes based on electromagnetic, structural mechanics, acoustics, fluid flow, heat transfer, and chemical engineering behavior. It is the finite element software commonly used in engineering calculations [24].

**Figure 8:**
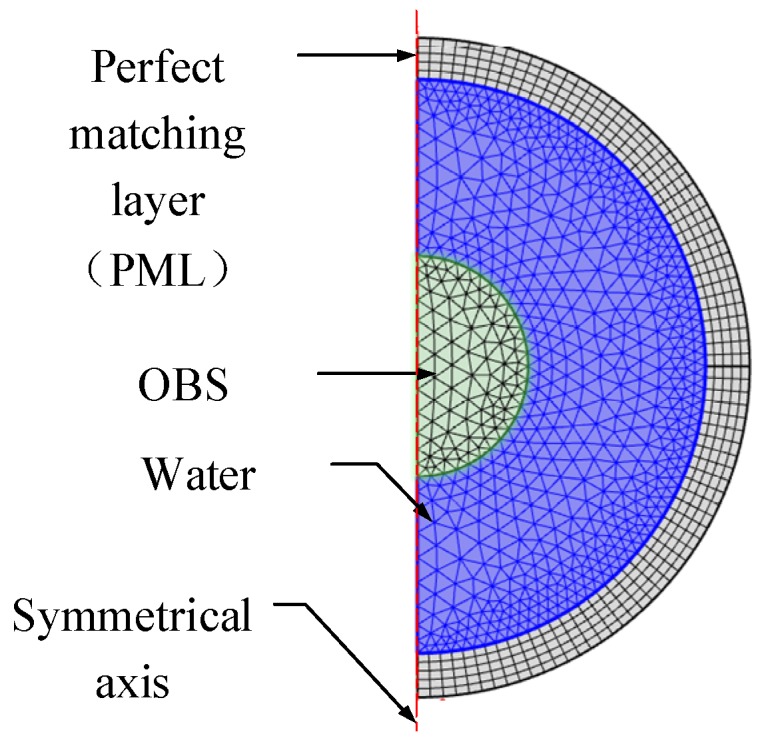
Axial symmetry model of the OBS in free field.

Figure 8 represents our indigenously built model for calculations of the vibration velocity and the scattering intensity. COMSOL software help us to build this model. In this model, the OBS is an iron sphere with a radius of 250 mm. The water layer around the OBS is wrapped with a width of 350 mm. The outermost layer is a perfect matching layer (PML) of 0.15 m in width. In this model, the incident acoustic wave is a plane wave with a frequency of 1 Hz~10 kHz and its amplitude is kept as 1 Pa. The characteristics of the individual components of OBS model in free field are given in Table 2. 

**Figure 9:**
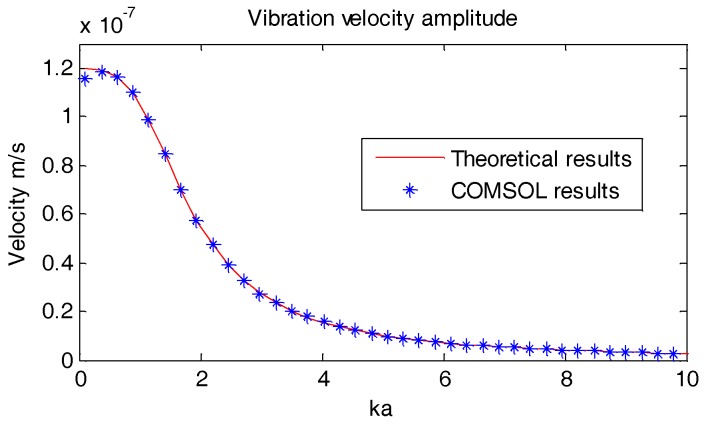
Vibration velocity amplitude of the OBS in free field.

**Figure 10:**
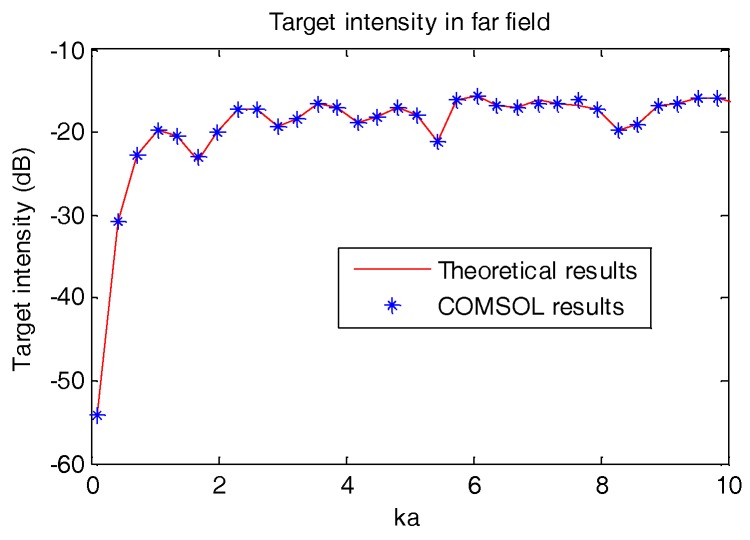
Scattering intensity of the OBS in free field.

Figure 9 and Figure 10 below display the vibration velocity and the scattering intensity of the OBS which are calculated using the COMSOL software. 

$$\;\mathrm{ka}\;=\;\frac{\omega }{c}a\;=\;\frac{2\pi f}{c}a\;$$

For both Figure 9 and Figure 10, the *ka* in *x*-axes is the product of wave number *k* and radius of the OBS *a*. The *y*-axes for both figures are vibration velocity (m/s) and scattering intensity (dB@1μpa) of the OBS in free field respectively. In the above equation, *f* is the frequency and *c* is the underwater sound speed. It can be concluded that the theoretical results of the vibration velocity amplitude and the scattering intensity of the OBS in free field are in accordance with that calculated by COMSOL. This proves the authenticity of our model and thus can be employed to calculate the scattering intensity and vibration velocity of OBS in free field.

**Figure 11:**
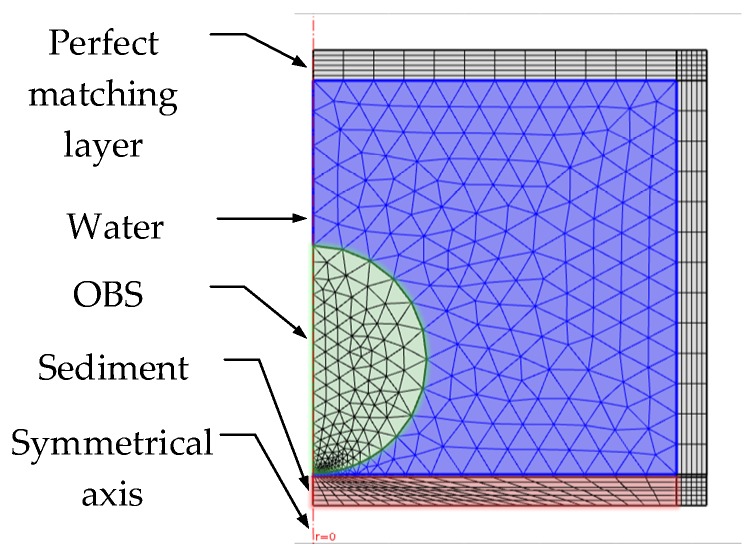
Axial symmetry model of an OBS placed on the seafloor.

Then, as is shown in Figure 11, a new model with the OBS placed on the seafloor sediment is built in COMSOL. In this new model, the OBS is of same size as previous model, and surrounded by water. The model is covered from outside with a perfect matching layer (PLM). Assuming that the water layer and sediment in model were both homogeneous media, the sediment is fluid layer. The OBS is a solid iron sphere with characteristics summarized in Table 2 and Table 3. The incident wave is the plane wave with a frequency of 100 Hz~10 kHz and amplitude of 1 Pa.

To verify the accuracy of this particular model, the angle *β* of incident wave is set as 0°. In this way there will be only horizontal incident plane wave without any reflection from the seafloor. In Equation (9), the seafloor reflection coefficient *R*(*β*) will become zero, the vibration velocity of the OBS placed on the seafloor should be similarly with the velocity in free field.

**Figure 12:**
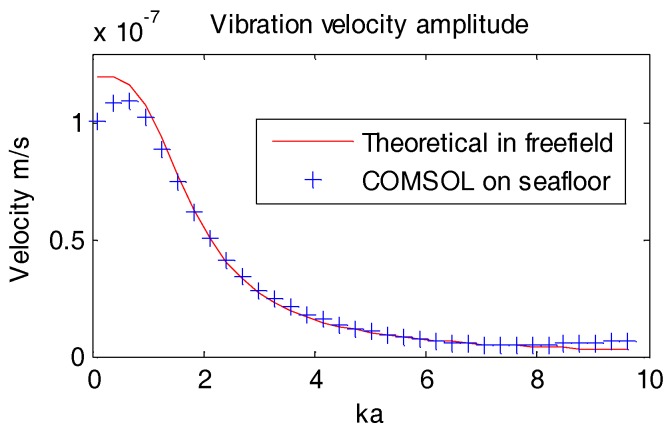
Vibration velocity amplitude of the OBS under incident angle of 0°.

As presented in Figure 12, the COMSOL calculations for the vibration velocity amplitude of the OBS placed on seafloor under horizontal incident wave is equal to that of the OBS in free field. It proves that our new COMSOL model is also capable of calculating the vibration velocity for the OBS placed on seafloor.

## 4. Vibrational Response of the OBS

The conclusions in Section 2 suggest that the vibration velocity amplitude of the OBS placed on seafloor is dependent upon the OBS shape, physical parameters (density, P-wave speed, S-wave speed), and structure. In the following sections, calculations shall be done by our COMSOL model. These calculations will measure vibration velocities of OBSs made up of varying densities, shapes, and characters. The results, in turn, will reduce the vibration amplitude excited by underwater noise.

### 4.1. Vibration Velocities of the OBS with Varies Density Exposed to the Underwater Noise

A new COMSOL model is considered here with an OBS of 75 mm in diameter. This model is based on the previous model (Figure 11) with only reduced size of the OBS. The volume of this small size OBS is of standard size comprising batteries, sensors, and electronic circuits. The parameters of this newly developed model are same as mentioned in Table 2. The vibration velocity amplitudes for three OBSs of same spherical shape but having various materials (Table 4) i.e., iron, resin, and aluminum. They are measured against frequency (1 Hz~1 kHz). In addition, all the measurements are done with varying incident angles of underwater acoustic waves such as 10°, 60°, and 90°.

**Figure 13:**
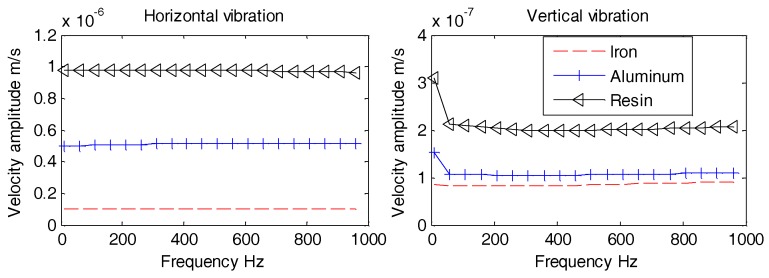
Vibration velocity amplitudes of the OBSs with different densities under incident angle of 10°.

**Figure 14:**
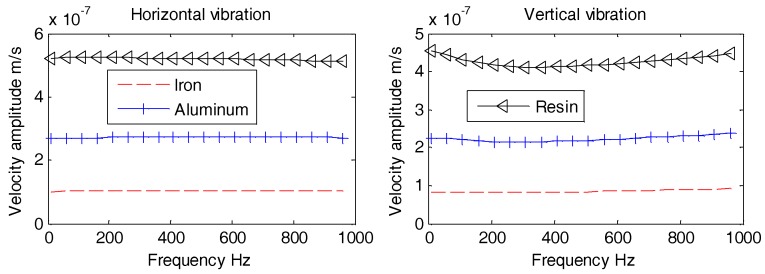
Vibration velocity amplitudes of the OBSs with different densities under incident angle of 60°.

**Figure 15:**
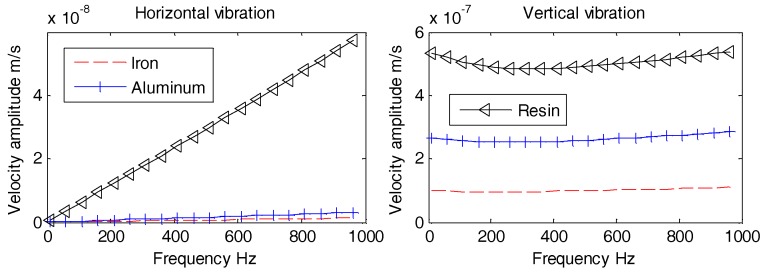
Vibration velocity amplitudes of the OBSs with different densities under incident angle of 90°.

The results obtained in Figure 13, Figure 14 and Figure 15 suggest that all OBSs experience smoother horizontal and vertical vibration velocity amplitudes without large fluctuations. The horizontal and vertical vibration velocity amplitudes reduce with the increase in the density of the OBS manufacturing material. Thus, we may conclude that the OBS with higher density has lower vibration velocity amplitude especially under the influence of underwater noise. However, according to the calculations in Reference [25,26], the vibration velocity amplitude of the OBS will reduce as the density increases under seismic waves as well. Thus, the average density of the designed OBS should be consistent with the density of seafloor, which can reduce the effect of the underwater noise and at the same time, may achieve better perception of seismic waves. For that purpose, OBS should be placed and worked at seafloor with relatively hard surface having higher density.

### 4.2. Vibration Velocities of the OBSs with Various Shapes Exposed to the Underwater Noise

**Figure 16:**
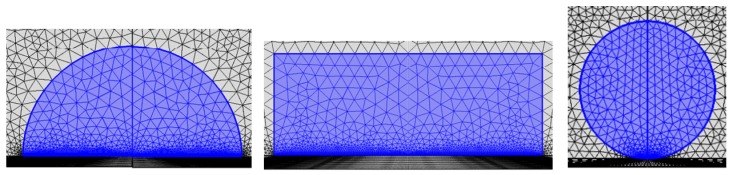
The OBSs with various shapes.

Common OBSs can be classified into spherical, cylindrical, and hemispherical types (Figure 16) depending on their physical appearances. In this section, the vibration velocities of the OBSs with various shapes but having similar volumes and average densities of 1800 kg/m^3^, will be calculated against various incident angles.

**Figure 17:**
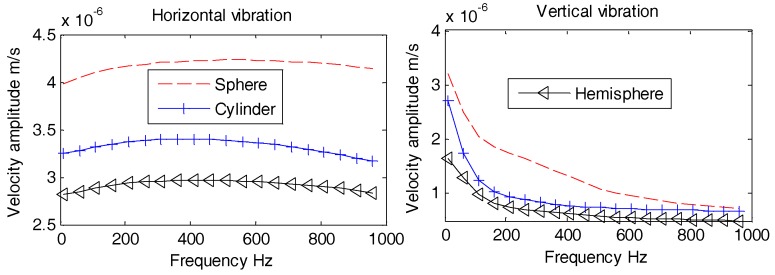
Vibration velocity amplitudes of the OBSs with different shapes under incident angle of 10°.

**Figure 18:**
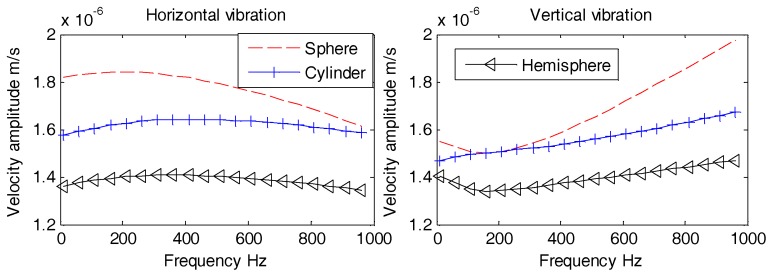
Vibration velocity amplitudes of the OBSs with different shapes under incident angle of 60°.

**Figure 19:**
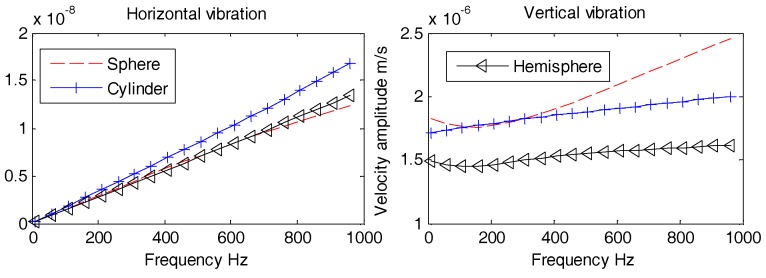
Vibration velocity amplitudes of the OBSs with different shapes under incident angle of 90°.

Aforementioned Figure 17, Figure 18 and Figure 19 reveal that all horizontal and vertical vibration velocity curves are quite flat with the exception of the spherical OBS. The vibration velocity amplitudes of the spherical OBS are observed to be the largest, regardless of the angle of incident waves.

We may conclude that in order to designs an OBS with lowest amplitude of vibration velocity, the hemispherical shape is the best option. The hemispherical OBS is also capable of not only preventing fishing trawler nets but also reduces the induced flow noise. The development of hemispherical outer casing is not only complex technologically but is expansive as well. Hence, it is not suitable for mass production. For this particular reason, development of a cylindrical OBS is preferred despite knowing the fact that its vibration velocity amplitudes are a bit higher than the hemispherical one. In addition, development and manufacturing of the cylindrical OBSs are relatively simple, low cost, and also suitable for mass production.

### 4.3. Vibration Velocities of the OBSs with Different Internal Compartments Exposed to the Underwater Noise

**Figure 20:**
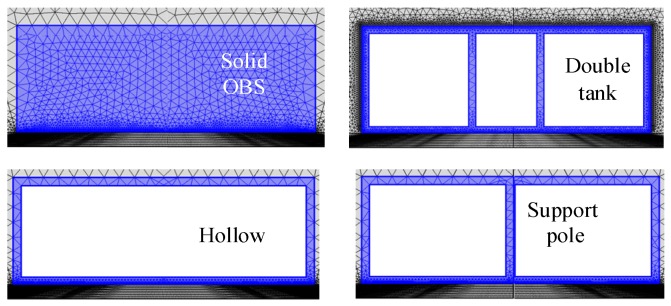
The cylindrical OBSs with same average density but different internal compartments.

The interior part of OBS is kept hollow in order to place the batteries, circuit boards, and vibration sensors. The vibration response of hollow OBS is complicated than that of solid one. In this section, the vibration velocities of cylindrical OBSs made up of iron with various configurations of internal compartments are analyzed. The various configurations of internal compartments are solid, hollow, support pole, and double-tank as shown above in Figure 20.

**Figure 21:**
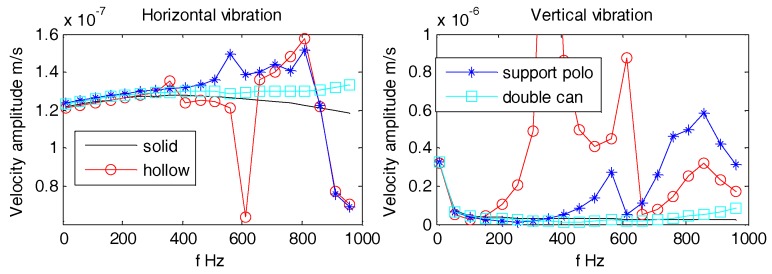
Vibration velocity amplitudes of OBSs with different compartments under incident 10°.

**Figure 22:**
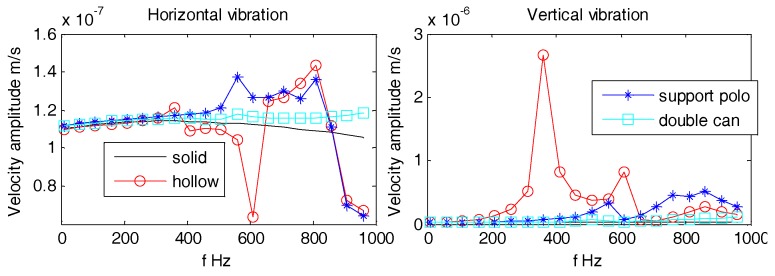
Vibration velocity amplitudes of OBSs with different compartments under incident 60°.

**Figure 23:**
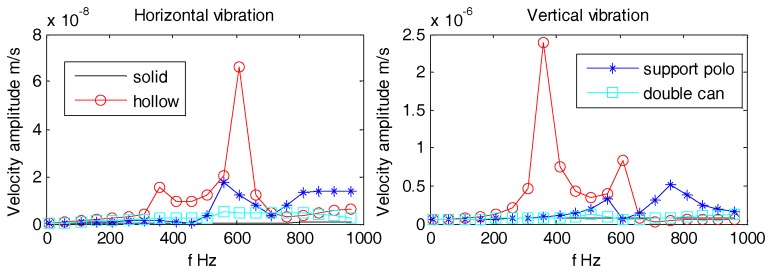
Vibration velocity amplitudes of OBSs with different compartments under incident 90°.

Aforementioned Figure 21, Figure 22 and Figure 23 suggest that, regardless of the incident angle, all the amplitudes are stable with no difference at lower frequencies (1 Hz~100 Hz). The horizontal and vertical vibration velocity amplitudes of hollow-structure OBS display abrupt changes at higher frequencies (100 Hz~1 kHz). The vibration velocity amplitudes for both horizontal and vertical directions of support pole OBS especially at center exhibit few fluctuations. These fluctuations are slightly lower in amplitudes than those of the hollow OBS. The Double-tank OBS has shown characteristics quite similar to that of the solid one since its vibration velocity amplitude remained stable for most of the time. We may conclude now that development of a double tank OBS is preferred over the hollow structure OBS. The double tank design offers added advantage of placing vibration sensors in the inner tank, which effectively reduces the interference of electromagnetic radiations caused by the batteries and/or circuits. 

### 4.4. Vibration Velocities of the OBSs with Different Density Gradients Exposed to the Underwater Noise

Due to the limitation in the internal spacing of the OBS, vibration sensors, acquisition circuits, and batteries need to be placed in a precise and efficient way. The battery pack is heavier than the circuit boards. This makes the OBS quite imbalanced especially in the vertical direction. The OBS with various weight imbalances experience various vibration velocities especially when influenced by the underwater noise. In this forthcoming section, vibration velocities of the OBSs with various density gradients will be investigated and analyzed accordingly.

**Figure 24:**
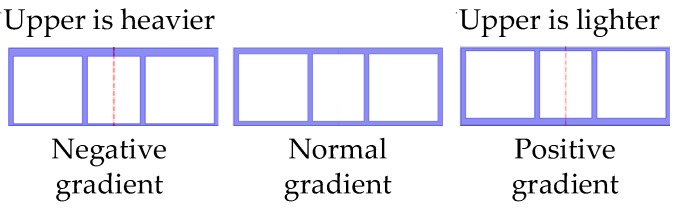
The OBSs with varying density gradients.

Firstly, we assume that the density gradient of the OBS is positive gradient when the OBS battery pack is placed near the bottom, which means that OBS is lighter at surface and heavier at bottom. On the contrary, density distribution of OBS is called negative gradient when surface is heavier and the bottom is lighter in weight (both positive and negative gradient configurations are shown in Figure 24). Finally, the normal gradient exhibits the equal weights on both surface and bottom of OBS, as is shown in Figure 24.

**Figure 25:**
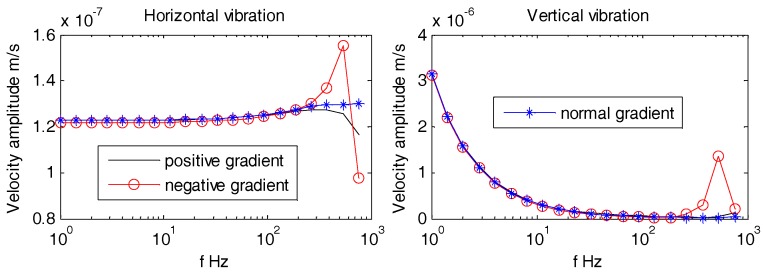
Vibration velocity amplitudes of the OBS with density gradients under incident angle of 10°.

**Figure 26:**
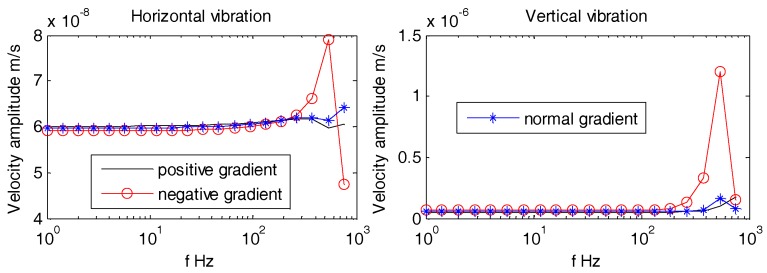
Vibration velocity amplitudes of the OBS with density gradients under incident angle of 60°.

**Figure 27:**
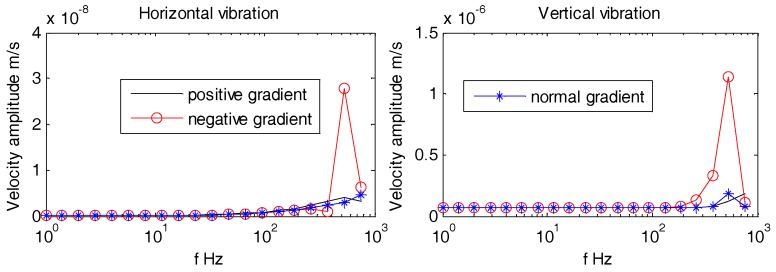
Vibration velocity amplitudes of the OBS with density gradients under incident angle of 90°.

As can be seen from Figure 25, Figure 26 and Figure 27, the density gradients of OBS has little effect on the vibration velocity amplitudes at lower frequencies (1 Hz~100 Hz). When the frequency approaches 1 kHz, the vibration velocity amplitudes of the OBSs with negative gradient are large, but the amplitudes are small and have little difference in case of both the positive and normal gradient OBSs.

Thus, we may conclude that in case of designing low frequency (<100 Hz) OBSs, the influence of density gradient plays no role whatsoever. For high-frequency OBS, the design with positive density gradient should be adopted as much as possible, which can reduce the influence of noise on OBS in water. In addition, the positive gradient OBS placed on the seafloor for detection of seismic waves performs relatively better [26].

### 4.5. Vibration Velocities of OBSs with Different Outer Casings Exposed to the Underwater Noise

**Figure 28:**
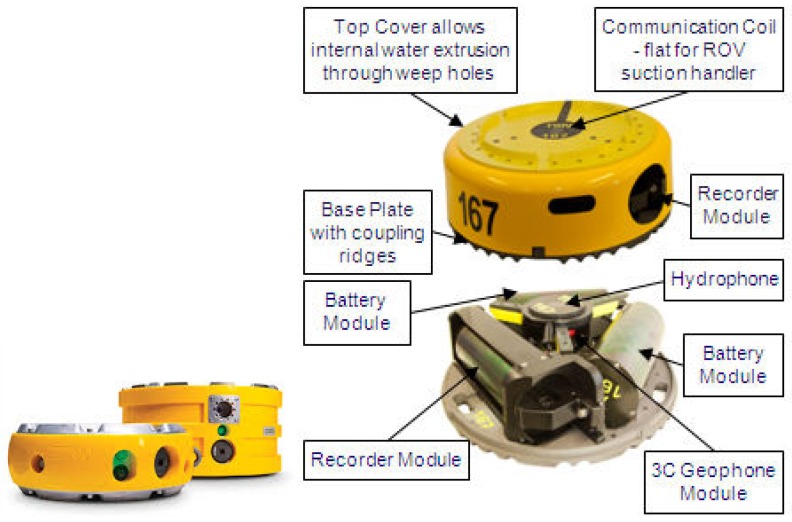
The OBSs with various outer casings.

**Figure 29:**
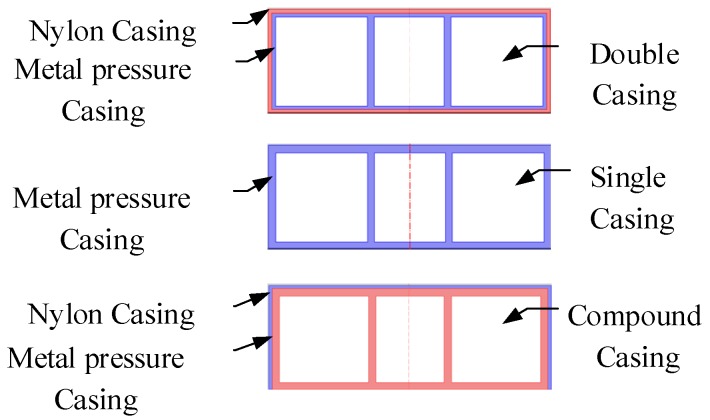
The OBSs with different casings.

Usually, the OBSs have relatively smooth, regular, and non-metallic outer casing i.e., Z700 etc., as shown in Figure 28. Figure 29 displays multi-variety casings for the pressure tank. The vibration velocities of the OBSs with multi-variety casings are analyzed in the coming section.

**Figure 30:**
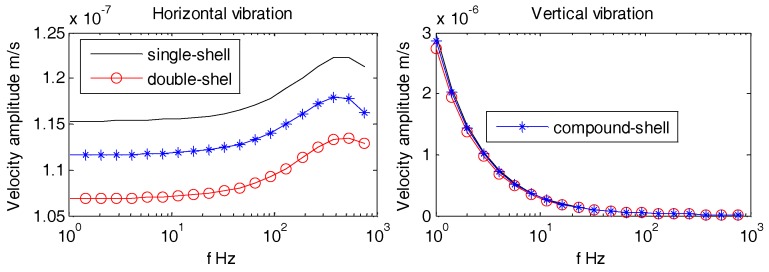
Vibration velocity amplitudes of the OBSs with various casings under incident angle of 10°.

**Figure 31:**
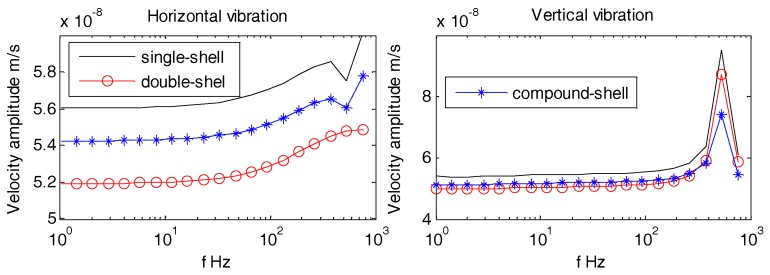
Vibration velocity amplitudes of the OBSs with various casings under incident angle of 60°.

**Figure 32:**
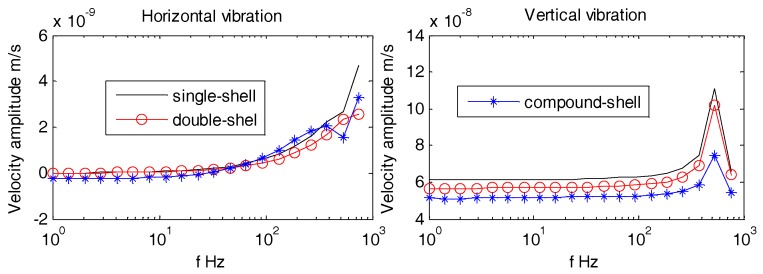
Vibration velocity amplitudes of the OBSs with various casings under incident angle of 90°.

Figure 30, Figure 31 and Figure 32 suggest that the horizontal and vertical vibration velocities for various outer casings exhibit negligible changes. At lower frequencies (1 Hz~100 Hz), the amplitudes of all the OBSs are relatively stable, so the design of outer casing for the OBS doesn’t matter for lower frequencies. For higher frequencies, the outer casing design shall be dependent on the relevant applications

## 5. Experimentation in the Real-Sea Environment

**Figure 33:**
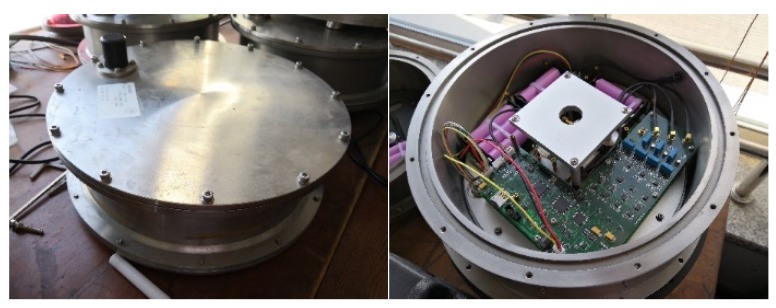
Design of a cylindrical double-tank OBS made up of titanium alloy.

**Figure 34:**
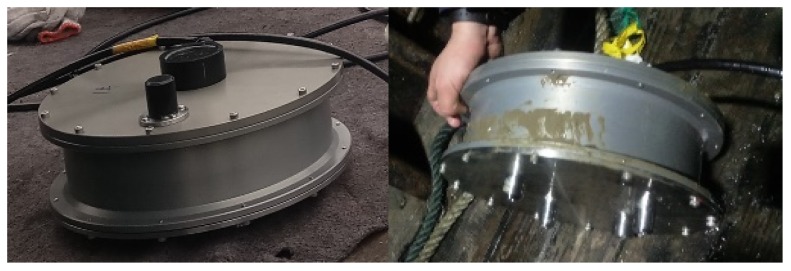
The OBS before deployment to the seafloor and just after the recovery.

The cylindrical double-tank OBS was applied for the experiment in order to measure the high frequency seismic waves (100 Hz~1 kHz), as shown in Figure 33 and Figure 34. The body of the OBS was manufactured by using TC-4 titanium alloy with an average density of 1.8 kg/m^3^. The vibration velocity sensors were set in the interior tank of OBS, while the batteries were installed in a periphery close to the bottom.

**Figure 35:**
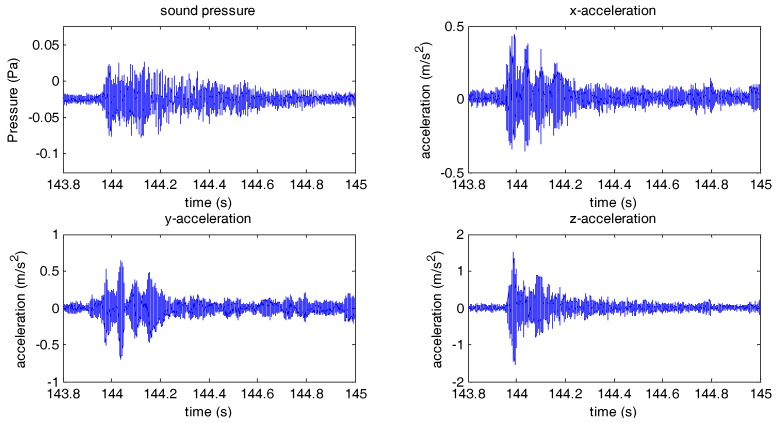
The sound pressure and vibrational signal received by the OBS.

The OBS was deployed nearly 10 km away from the east coast of Qingdao with negligible sea traffic. Our ship was moored at about 10 km away from the east of OBS under experimentation. Explosives were deployed from our moored ship and programmed to blast in the middle of seawater. The OBS under experimentation was capable of receiving the seismic waves generated by explosive sources. The sound pressure and vibration in three directions received by the OBS are illustrated in Figure 35 below.

**Figure 36:**
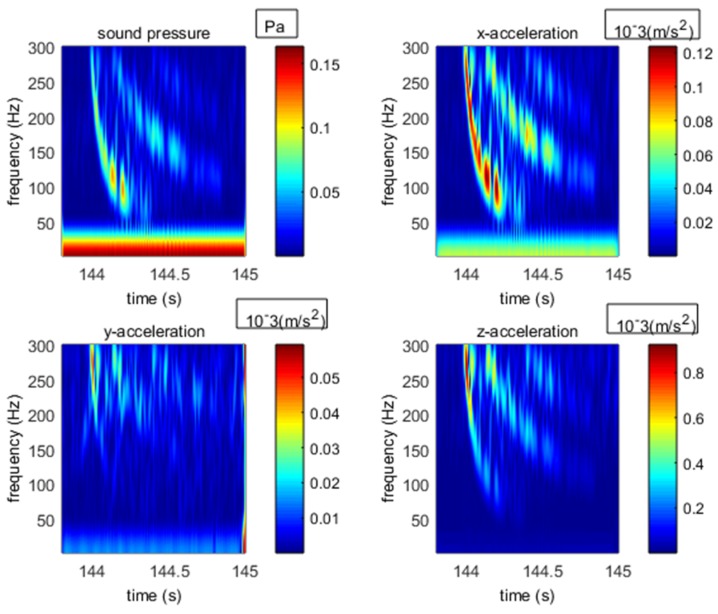
**Figure36.** The time-frequency analysis for different channels.

**Figure 37:**
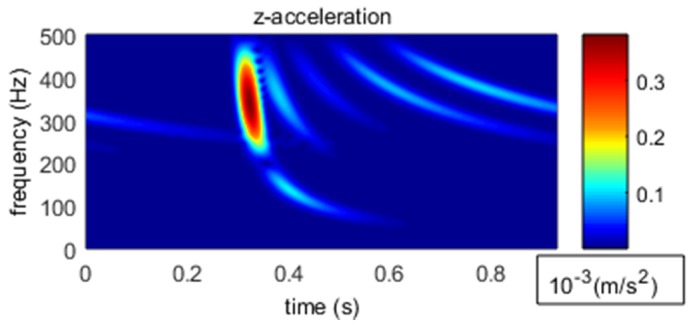
Time-frequency analysis of simulated seismic waves in shallow water.

In shallow water, both broadband acoustic and seismic waves experience different group velocities at different frequencies. This phenomenon of varying group velocities with varying frequencies is termed as frequency dispersion. Time-frequency analysis is an important method to analyze the frequency dispersion, as shown in Figure 36 [15] for simulation results and in Figure 37 for our measured results. As shown in Figure 37, the arrival time of seismic waves at different frequencies were asymmetrical which clearly demonstrated the frequency dispersion. 

Figure 36 displays the dispersion phenomenon for the time-frequency analysis of the signal received by our OBS. It indicates that our OBS can accurately detect the broad-band seismic waves radiated by explosive sources. In addition, the energy distribution of our measured results at each frequency agreed with the laws of sound propagation in shallow water. This real ocean experimental activity proved that our designed OBS is capable of collecting high frequency signal (100 Hz~1 kHz) both accurately and effectively.

## 6. Conclusions

In this paper, the sound field on the surface of OBS and its vibration velocity is deduced in the presence of a seafloor interface. COMSOL is used to build the vibration response model for the OBS exposed to low frequency (1~1 kHz) noise in the water. Furthermore, the vibration velocities of OBSs with different densities, shapes, and structures are compared and analyzed. 

The major contributions of this study are as follows:

(1) The OBS with same shape and structure, but with heavier body, experience lower vibration velocity amplitudes for signal of either seismic waves or underwater noise. The average densities for both OBS and the sea-floor need to be similar at the experimentation site. A rocky seafloor with larger density is preferred for appropriate working of OBS.
(2) Due to low ocean water pressure in shallow water, a cylindrical OBS is s used for our experimentation activity. Though the vibration velocity amplitudes of the hemispherical OBS is lower especially when it is exposed to underwater noise, but its overall design and development are quite expansive and complex. Thus the only option is cylindrical OBS during the experimentation in real-sea environment.
(3) Designing the OBS for lower frequencies (<100 Hz) doesn’t take into account the configuration, density gradient, nor the type of outer casing.
(4) For higher frequencies (>100 Hz), recommended design for development of OBS is in double-tank configuration. This design should be positive gradient with compound outer casing in order to reduce the vibration velocity amplitudes especially when the OBS is exposed to underwater noise.

To summarize, the cylindrical OBS with consistent average density seafloor, double tank, density in positive gradient, and compound outer casing has relatively minimum amplitudes of vibration velocity under the effect of the noise (1 Hz~1 kHz) in water. The given COMSOL model provides a fast and accurate method for minimizing the influence of underwater noise.

## Figures and Tables

**Table 1:** Comparative of present Ocean Bottom Seismometers (OBS) [18].

Model	WHOI Hole-D2	IRD-UTIG	Micro OBS	I-7C
Nationality	USA	USA	Germany	China
Channels	4	4	3	7
Release	electrolytic	electrolytic	electrolytic	mechanic
Consume (W)	1	0.5	0.6	0.25
Autonomy (h)	60	60	10	120
Weight (kg)	73	85	10	45
Height/width/length (m)	1 × 1 × 0.56	0.6 × 1 × 1	0.4× 0.4 × 0.4	1.5 × 1.5 × 0.8

**Table 2:** Material parameters in the free field.

Material	Density (kg/m^3^)	P-Waves (m/s)	S-Waves (m/s)
Water	1000	1500	0
PML	1000	1500	0
OBS	7850	5848	3233

**Table 3:** Material parameters of sediment.

Material	Density (kg/m^3^)	P-Wave (m/s)	S-Wave (m/s)
Sediment(silt)	1800	1600	0

**Table 4:** Density of the different materials.

Material	Density (kg/m^3^)	P-Waves (m/s)	S-Waves (m/s)
Iron	7850	5848	3233
Resin	1180	2695	1100
Aluminum	2700	6198	3122
